# The Dietary Carbohydrate/Fat-Ratio and Cognitive Performance: Panel Analyses in Older Adults at Risk for Dementia

**DOI:** 10.1016/j.cdnut.2023.100096

**Published:** 2023-05-07

**Authors:** Jakob Norgren, Shireen Sindi, Anna Sandebring-Matton, Tiia Ngandu, Miia Kivipelto, Ingemar Kåreholt

**Affiliations:** 1 Division of Clinical Geriatrics, Center for Alzheimer Research, Department of Neurobiology, Care Sciences and Society, Karolinska Institutet, Stockholm, Sweden; 2 Neuroepidemiology and Ageing Research Unit, School of Public Health, Imperial College London, London, United Kingdom; 3 Division of Neurogeriatrics, Center for Alzheimer Research, Department of Neurobiology, Care Sciences and Society, Karolinska Institutet, Stockholm, Sweden; 4 Population Health Unit, Department of Public Health and Welfare, Finnish Institute for Health and Welfare, Helsinki, Finland; 5 Theme Inflammation and Aging, Medical Unit Aging, Karolinska University Hospital, Stockholm, Sweden; 6 Stockholms Sjukhem, Research and Development Unit, Stockholm, Sweden; 7 Institute of Gerontology, School of Health and Welfare, Aging Research Network–Jönköping, Jönköping University, Jönköping, Sweden; 8 Aging Research Center, Department of Neurobiology, Care Sciences and Society, Karolinska Institutet and Stockholm University, Stockholm, Sweden

**Keywords:** macronutrients, cognitive decline, memory, aged, compositional data, nutritional epidemiology, target trial

## Abstract

**Background:**

Roughly 80% of total energy intake (TEI) in most human diets originates from digestible carbohydrates (eCarb) and fat (eFat), but the impact of their proportions on cognitive performance is poorly understood.

**Objectives:**

Our primary aim was to investigate estimates of global cognition in relation to macronutrient intake, with the log-ratio eCarb/eFat (CFr) as the primary predictor variable of interest. Secondary predictors were protein and the saturated/total fat ratio. Exploratory comparisons of CFr with eCarb and eFat as separate predictors were an additional aim.

**Methods:**

The observations were made on panel data (years 0, 1, 2) from the Finnish Geriatric Intervention Study to Prevent Cognitive Impairment and Disability, *n* = 1251; age 60–77 y; 47% females; selected by risk factors for dementia. Self-reported diet was assessed by 3-d food records. Global cognition was measured using a modified Neuropsychological Test Battery. A mixed linear regression model was used, adjusted for age, sex, education, body-mass index, cholesterol-lowering drugs, TEI, time, time × intervention/control group, with study site and subject as random factors. Estimates were standardized (mean = 0; SD = 1) with 95% CI.

**Results:**

CFr had a negative estimate to global cognition (*β* = −0.022, CI: −0.039, −0.005; *P* = 0.011). The point estimate for protein was *β* = 0.013 (*P* = 0.41), and for the saturated/total fat ratio, associations with cognition were nonlinear. CFr correlated highly with eCarb (Pearson’s *r* = 0.92) and eFat (*r* = −0.94). The point estimate for CFr fell between eCarb (*β* = −0.026, *P* < 0.001) and (inversely) eFat (*β* = 0.017, *P* = 0.090).

**Conclusions:**

A lower CFr was associated with better global cognition among older adults at risk for dementia. Because this is an important target group for preventive interventions, clinical trials are warranted to further investigate the impact of macronutritional composition on cognitive health. The potential role of CFr as a predictor for cognitive health should be further studied.

## Introduction

The maintenance of cognitive health in older age—that is, the prevention of cognitive decline and dementia—may be promoted by dietary factors [1,2]. The association between diet and cognitive health has been studied with various approaches, including single foods or micronutrients, dietary patterns, and macronutritional composition [3]. Macronutrients have the potential to be burned as fuel, but the modifiable range of their proportional contribution to total metabolized energy (measured as E%; prefix e) may exceed 10–75 E% for total digestible carbohydrates (eCarb) and total fat (eFat) while being <1 E% for some omega-3 PUFA [[4], [5], [6]]. The modifiable range of protein (eProt) is substantially lower compared with eCarb and eFat and exhibits lower variability within populations [7]. Typically, eProt in human diets supplies ≈10–20 E% [8] and ≈35 E% is assumed to represent a tolerance limit for humans [9]. The heterogenous category fiber (eFib) includes carbohydrates that cannot be absorbed in the intestine but may be converted to SCFAs by microbes in the colon [10], and humans typically extract only ≈2 E% from fiber [11]. Alcohol (eAlc) is an optional energy contributor that should not exceed 5 E% according to the Nordic Nutrition Recommendations (NNR) [12].

By distinct metabolic pathways [13], eCarb, eFat, eProt, eFib, and eAlc represent main energy categories that can be divided into subcategories, for example, SFA, MUFA, PUFA, sugars, starch, and specific amino acids*.* Because of the relatively narrow modifiable range for eProt (+ eFib and eAlc), eCarb and eFat constitute our predominant fuels and sum up at ≈80 E% in broad human diets [4,8]. Whereas the proportions of eCarb/eFat may be studied for a hypothesized shift in the general metabolic state, for example [5,14], the importance of some subcategories rather relates to specific structural or signaling functions [15,16], whereas their energetic contribution might be negligible, making them allied to micronutrients [6]. Macronutritional composition may influence factors related to cognitive health, for example, blood pressure, lipids, insulin, and weight [1,17].

Evidence on the impact of total fat or carbohydrate intake on cognitive health is inconsistent, for example, [[18], [19], [20], [21], [22], [23], [24]], and beyond the aims of this study to review in detail. A similar ambiguity extends beyond the cognitive field [25]. A synthesis of the evidence on macronutritional composition is complicated by the complexity of the topic, and by heterogenous methodology—which has been acknowledged as an area of improvement for nutritional epidemiology [26]. Notably, studies reporting subcategories of macronutrients far outnumber studies on eCarb and eFat in relation to cognitive decline [3] as well as to cognition in healthy adults [27]. We have not identified any study reporting eCarb/eFat as a ratio in relation to cognition, although it is suggested as a strategy to deal with collinearity among compositional data, that is, portions summing up to a whole [28].

The primary aim of these analyses was to investigate how global cognition is influenced by the proportional intake of carbohydrates and fat, with the log-ratio eCarb/eFat (CFr) as the primary predictor variable of interest. Secondary predictors of interest include eProt and the ratio saturated/total fat (SAFr). An additional, methodological, aim was to evaluate CFr as a predictor variable compared with individual reporting of eCarb and eFat.

## Methods

### Study design

Observational panel analyses were performed, with diet and cognition measured at 3 consecutive years. This data structure allows separated analyses on between- and within-subject effects in a way that is rarely reported in the nutrition field, but is common in biology [29], epidemiology [30], and social sciences [31]. Further clarification on how the 2-dimensional method was applied is given in relation to descriptions of the statistical methods below. All participants with data on diet and cognition at baseline were included, with a complementary sensitivity analysis including only participants with data from all time points.

### Study sample

This study is based on data collected within The Finnish Geriatric Intervention Study to Prevent Cognitive Impairment and Disability (FINGER), a multicenter randomized controlled trial (RCT) in older adults (*n* = 1259) without substantial cognitive impairment [MMSE, mean (SD) = 26.7 (2.0)], selected by the Cardiovascular Risk Factors, Aging, and Incidence of Dementia (CAIDE) risk-score (≥6) [32] and cognitive testing to represent a sample with increased risk for dementia [33]. The 2-y FINGER trial was the first multidomain (exercise, diet, cognitive training, monitoring of vascular risk factors) lifestyle intervention to report improved cognition in a sample at risk for dementia [34]: The group receiving intense intervention improved global cognition significantly more (Cohen’s *d* = 0.13) than the control group receiving regular health advice.

The dietary recommendations given to all participants—with counseling sessions by a dietitian for the active group—aimed at adherence to the Finnish Nutrition Recommendations at the time (based on NNR2004 [12]). The control group received similar health advice briefly. Food-level goals communicated to participants were translated into the FINGER dietary adherence score (FDAS), graded 0–9, to measure adherence [35]. Six components of the score were related to macronutrients: protein 10–20 E%, alcohol <5 E%, fiber >3 g/MJ, SFA + trans-fat <10 E%, PUFA 5–10 E%, and sucrose <10 E%. The remaining components targeted intake of fruit (≥200 g/d), vegetables (≥200 g/d), and fish (any intake). Analyses on changes in FDAS [35] and its relation to cognitive outcomes [36] have been published. Although macronutrient targets for total fat or carbohydrates were not the focus of the intervention, the counseling was based on the official guidelines [12] for eFat (25–35 E%) and eCarb (50–60 E%) at that time.

The FINGER trial was approved by the coordinating ethics committee of the Hospital District of Helsinki and Uusimaa and registered at clinicaltrials.gov: NCT01041989. Further details are available in publications on the study protocol [33], and the primary results [34].

No analyses within this article aim at evaluating the trial, and therefore reporting follows STROBE-nut [37].

### Dependent and independent variables

The primary outcome global cognition was calculated as the mean *z*-score from 14 subtests from a paper-and-pen–based modified Neuropsychological Test Battery (NTB) conducted by trained study psychologists. NTB included tests related to 3 cognitive subdomains: memory, executive function, and processing speed, which each generated a subcomposite score [38]. The NTB is a modified and extended version of a test battery shown to be sensitive to changes in early Alzheimer disease [39]. For diet variables where a significant association with global cognition was observed, exploratory analyses on cognitive subdomains were additionally performed.

**FIGURE 1:**
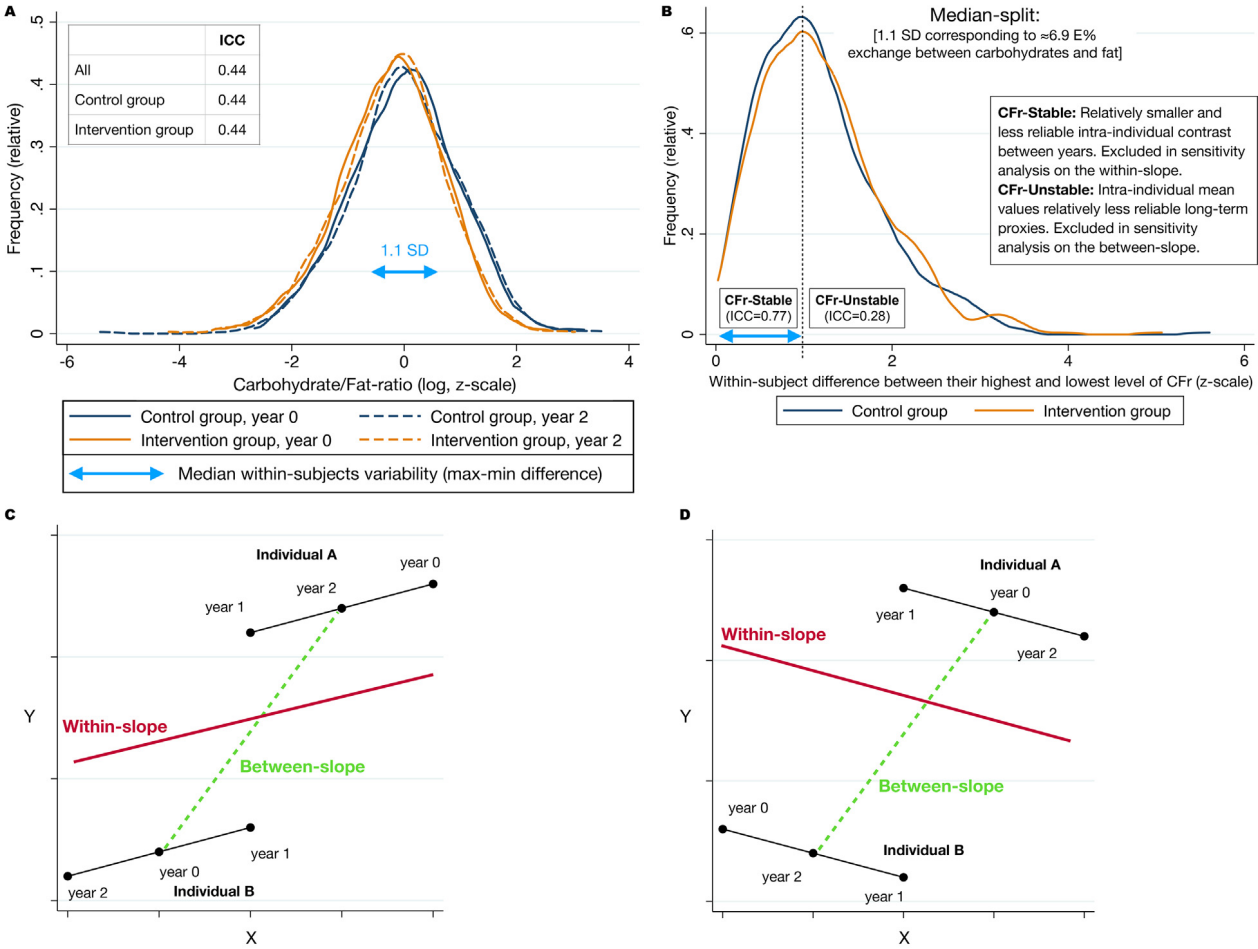
Methodological clarifications. (A) The homogenous distribution of CFr between randomization groups and time points is illustrated (*n* = 1029, matched between years). (B) Definition of CFr-Stable/unstable, showing that half of the participants differed by ≥1.1 SD between their highest and lowest measure of CFr (independent of order). The correspondence of 1.1 SD in CFr to 6.9 E% exchange between eCarb and eFat was estimated by crude regression reported below. (C) A hypothetical scenario for individual A and B when the between-slope (green/striped, generated from intraindividual mean levels of X and Y) and the within-slope (red/solid, “averaged” from everyone’s within-slope) are coherent in direction. (D) In contrast to panel C, the between and within slopes have opposing directions that might be a warning for unmeasured confounding. This could happen when the true effect of X on Y is detrimental but higher levels of X is typical for “health aware” people, which for other reasons have higher values of Y. CFr, log-ratio carbohydrates/fat; eCarb, carbohydrates by E%; eFat, fat by E%; ICC, intraclass correlation coefficient.

Values of the diet variables were collected from self-reported 3-d food records, checked by a study nurse, and recorded by trained nutritionists into computer software for analysis [35]. Potential intake from supplements was not included. All diet variables had their distributions in the same range regardless of randomization group (intervention compared with controls) or time point, as shown for CFr in Figure 1A (similar analyses for the other diet variables are shown in Supplemental Figure 1). The definitions of diet-stable/unstable and between/within-slopes—used for sensitivity analyses—are shown in Figure 1B–D.

Our primary predictor variable of interest was CFr, which was descriptively compared with eCarb and eFat individually. Secondary predictors of interest were eProt and saturated fat as a ratio of total fat (SAFr). SAFr correlated almost perfectly (*r* = 0.99) with the log-ratio eSFA/(eMUFA + ePUFA) and was chosen as the ratio of interest for its more intuitive interpretation. Diet variables were primarily analyzed as continuous variables.

Macronutrients by E% constitute compositional data (summing up to 100%, as opposed to unrestricted data) that put restrictions on their use in regression models due to collinearity [28]. Our variable selection pay attention to the fact that eCarb and eFat internally harbor the substantial part of the collinearity present among the portions eCarb + eFat + eProt + eFib + eAlc = 100%: CFr correlated highly with eCarb (*r* = 0.92) and eFat (*r* = −0.94), which in turn correlated inversely (*r* = −0.74) with each other and summed up to 80 E% (median; percentile 10–90: 74–84 E%). CFr had relatively high independence from eProt (*r* = −0.12), SAFr (*r* = −0.09), and total energy intake (TEI, standardized separately by sex to mean = 0 and SD = 1 for both females and males; *r* = −0.15). Technically, eProt represents protein compared with all other macronutrients, that is, mainly the pool of eCarb + eFat.

**FIGURE 2:**
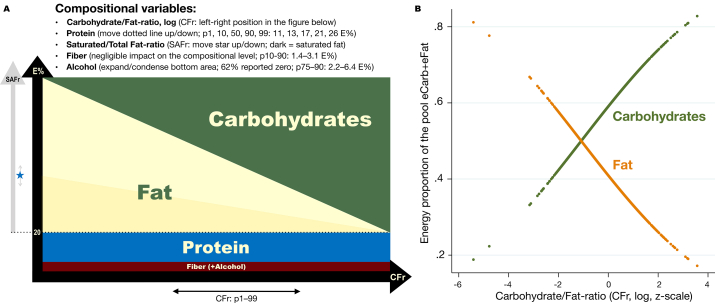
Visualization of macronutrient parameters. (A) The reciprocal relation between carbohydrates and fat and the distribution of their ratio (CFr) are illustrated. The substantially lower E% ranges of the other macronutrients at baseline are described. The sum of protein + fiber + alcohol is approximated to 20 E% in the figure. The actual distribution for p10, 25, 50, 75, and 90 was 16, 18, 20, 23, and 27 E%. (B) Visual validation of approximately interpreting CFr as “iso-caloric exchange between carbohydrates and fat within their internal pool.” CFr, log-ratio carbohydrates/fat; eCarb, carbohydrates by E%; eFat, fat by E%; p, percentile.

Pair-wise crude regressions indicated that 1 unit change for z_CFr_log (the standardized log-ratio) was associated with a change in eCarb of 6.31 (95% CI: 6.20, 6.41) E%, and with a change in eFat of −6.26 (−6.44, −6.09) E%, both *P* < 0.001. This gives that when the beta coefficient for z_CFr_log (that is, CFr) is divided by 6.3 in a reported model within this article, it can approximately be interpreted as change in the outcome (for instance cognition) from 1 E% exchange between carbohydrates and fat. For z_CFr_log = 0, the predicted levels of eCarb and eFat were 46.6 E% and 32.5 E%, respectively. Figure 2 is aimed at giving an intuitive interpretation of CFr in relation to the other diet parameters.

### Statistical analyses

In our study, both the independent (X) and dependent (Y) variables are considered reversible concepts (not disease-conversion) that are repeatedly measured, unlike all reported studies we have identified targeting macronutrients → cognitive health. As described in more detail by others [29,30], different approaches (A/B/C) can be chosen for analyzing such a data structure:

A: calculate mean X and mean Y of the repeated measures for each individual and make a pure between-subject analysis based on those collapsed means. This might be motivated if within-variability in X is assumed to reflect impression in measurement or random fluctuations rather than true changes in habits (X). Analyses do not take advantage of the information given by within-subject variability (STATA: xtreg, be).

B: make a regression X→Y solely based on within-subject variability in relation to intraindividual means of X and Y. This approach has an advantage of not being confounded by variables (including unmeasured) that are time-invariant within subjects [30]. Analyses do not take advantage of the information given by between-subject variability (STATA: xtreg, fe; “fixed effects model).”

C: combine strategies A and B by using a mixed effects model that integrates those components [29]. When reported alone, the slope may have an ambiguous interpretation: does it primarily reflect within- or between-effects? (STATA: mixed || subject).

Conceptually, A might capture a (relative) long-term effect of diet, whereas B more reflects potential effects of diet changes within the study period. For transparency, we report A, B, and C. The mixed model (C) was defined a priori as the primary result by assumptions that it most extensively takes advantage of the information within the data set. In Figure 1B–D, we illustrate how hypothetical patterns of A in relation to B and the exclusion of diet-stable/unstable respectively can guide further interpretations.

Complementary cross-sectional analyses were performed at baseline (per definition unbiased from the intervention) and at y1 and y2 (to visually explore the consistency of the associations). Clustering by study site (*n* = 6) and individual was adjusted for by including them as random factors in the models. Test for temporal lags [31] supported a model excluding lagged associations. Robust standard errors were estimated to reduce bias from potential heteroscedasticity.

A graphical check of linearity indicated no contraindications for using linear models, with 1 exception: because of an inversely u-shaped pattern for SAFr, it was reported by quintiles because linear estimates were not considered valid. A mild inversely u-shaped association with cognition was observed for TEI; although an added quadratic term was significant, it was dropped after having negligible effect on the results when used as a control variable.

All models were adjusted for time, and the interaction time × randomization group, to control for confounding by behavior changes and the known [34] changes in mean cognitive score along the study. After concluding that results were very similar when time was treated as a continuous compared with a categorical variable, it was used as a continuous variable. A 3-way-interaction time × group × diet_variable → global cognition was explored to assess potential differences between the intervention and control groups in the association between diet and cognition.

The crude model A had a descriptive purpose. Model B was additionally adjusted for the confounders age (years at baseline), sex, and education (y, truncated to 5–18 and log-transformed for a linear association with cognitive score). In model C, BMI (log-transformed) and statins (use of statins or other cholesterol-lowering drugs, yes/no) were added, primarily assumed to be confounders (although an interpretation as mediators might be possible). For comparability with model C, 4 individuals with missing data on BMI/statins were excluded in models A and B, giving a consistent sample size of *n* = 1247, with *n* = 1028 in the sensitivity analyses. Because TEI had some impact on the results and might have an alternative interpretation as a mediator rather than a confounder, model C is reported both with and without adjustment for TEI. Because adjustment for a mediator may increase bias for the predictor of interest [40] that was preferably avoided. Adjustment for the potential confounders smoking and diabetes had negligible impact, so they were not included in reported models. The diet variables eProt, SAFr, eFib, eAlc, and FDAS were exploratorily added 1 at a time to model C in the analyses of CFr. The following covariates were considered less likely confounders, or potential mediators, and were exploratorily reported as supplementary material: APOE-genotype, LDL-cholesterol, ApoB, the ratio triglycerides/HDL-cholesterol, fasting glucose, 2-h glucose concentration in oral glucose tolerance test.

The analyses were performed with STATA 15 software. β-Coefficients are reported with the predictor and outcome variables measured on a *z*-scale, unless otherwise specified. A significance level of *P* < 0.05 was used and 95% CIs are reported. Numerical reporting is excluded for exploratory or complementary results when reported graphically. Results are reported without any adjustment for multiple comparisons, which should be considered in the interpretation of those analyses referred to as exploratory or complementary [41].

## Results

### Participants

These analyses included 1251 participants with available baseline data on diet and cognition. Complete data on diet and cognition from all time points was available for *n* = 1029. A sample of *n* = 947 additionally had complete data on prioritized covariates. Baseline characteristics are described in Table 1.

**TABLE 1:** Baseline characteristics of the participants

	All available	Subsample (*n* = 947)
Age (y)	69.3 ± 4.7	69.1 ± 4.7
Female/male (*n* = 584/667)	47/53%	47/53%
Education (y)	10.0 ± 3.4	10.0 ± 3.4
Global cognition (*z*-score)	0.0 ± 1.0	0.06 ± 1.0
Intervention/control group	50/50%	48/52%
Diabetes (*n* = 152/1250)	12%	13%
Cholesterol-lowering drugs (*n* = 532/1208)	44%	46%
APOE ≥1 ***ε***-4 (*n* = 385)	31%	31%
APOE no ***ε***-4 (*n* = 781)	62%	63%
APOE unknown (*n* = 85)	7%	6%
BMI (kg/m^2^; *n* = 1242)	28.2 ± 4.7	28.2 ± 4.7
Fasting glucose (mmol/L; *n* = 1248)	6.1 ± 0.9	6.1 ± 0.9
Total cholesterol (mmol/L; *n* = 1247	5.2 ± 1.0	5.2 ± 1.0
HDL-C (mmol/L; *n* = 1247)	1.4 ± 0.4	1.5 ± 0.4
LDL-C (mmol/L; *n* = 1247)	3.1 ± 0.9	3.1 ± 0.9
Triglycerides (mmol/L; *n* = 1247)	1.4 ± 0.6	1.4 ± 0.6
ApoA-I (mmol/L; *n* = 1247)	1.6 ± 0.3	1.6 ± 0.3
ApoB (mmol/L; *n* = 1247)	0.9 ± 0.2	0.9 ± 0.2
TEI (kJ)	7810 ± 2277	7886 ± 2279
Carbohydrates (E%)	46.4 ± 7.1	46.5 ± 6.9
Total fat (E%)	32.4 ± 6.4	32.4 ± 6.3
Protein (E%)	16.9 ± 3.1	16.9 ± 3.1
Fiber (E%)	2.3 ± 0.7	2.3 ± 0.7
Saturated/total fat (%)	37.3 ± 6.2	37.1 ± 6.1

*n* = 1251, unless stated otherwise. Values as percentages or mean ± SD. The right column shows characteristics of a subsample used for complementary analyses, indicating that differences from the full sample were only marginal. APOE, apolipoprotein E (genotype).

### Distributions and correlations of the diet variables

**FIGURE 3:**
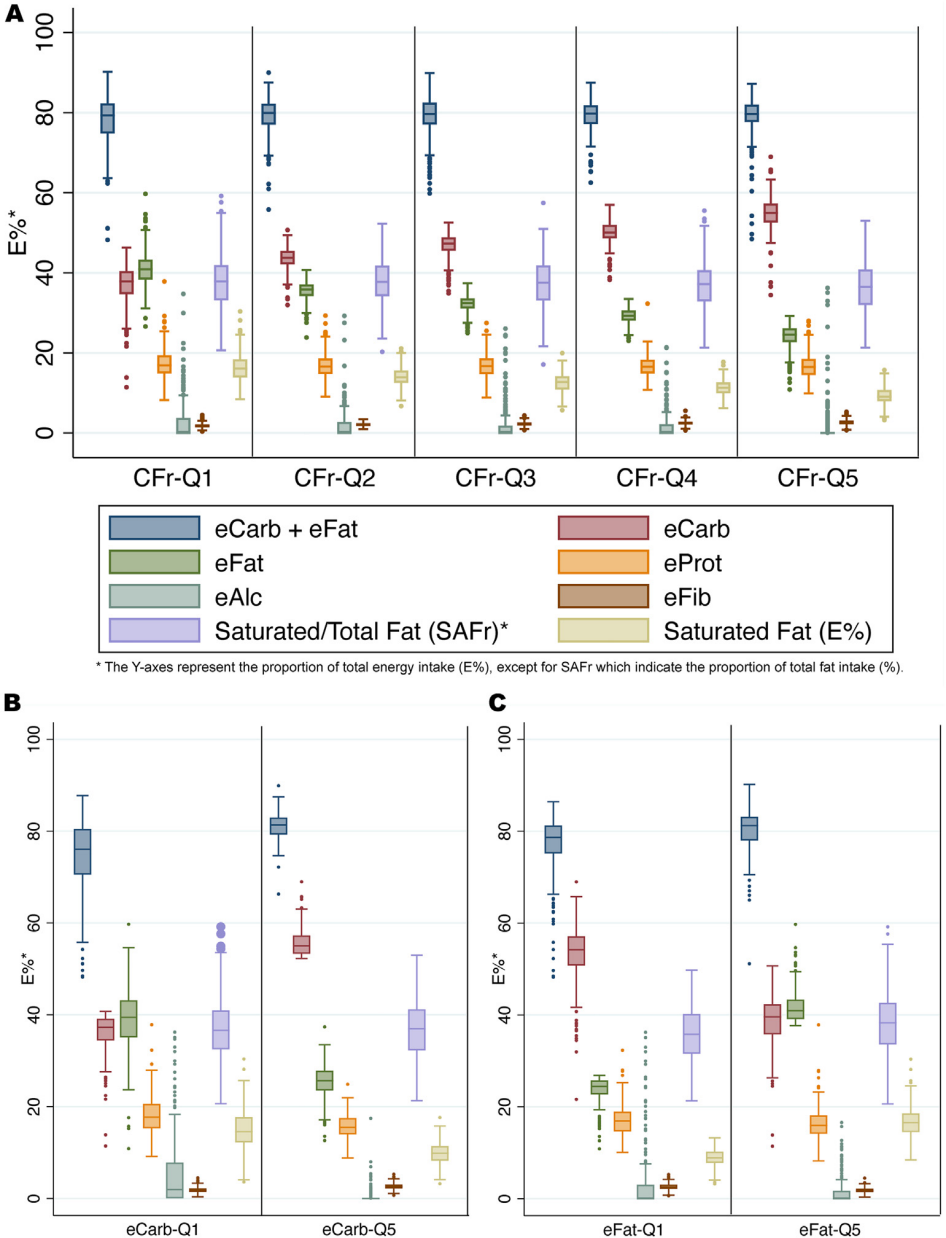
Distribution of macronutrients within quintiles based on CFr, eCarb, and eFat. (A) The relatively balanced baseline distribution of eProt and SAFr over CFr quintiles is illustrated. In contrast, eCarb (B) and eFat (C) are more confounded by outliers in eAlc and (at least for eCarb) eProt. The variables eCarb and eFat are shown to be proxies for CFr by having (inversely) similar distributions of eCarb and eFat. Boxes indicate percentiles 25, 50, and 75. CFr, log-ratio carbohydrates/fat; eAlc, alcohol (E%); eCarb, carbohydrates by E%; eFat, fat by E%; eFib, fiber by E%; eProt, protein by E%; SAFr, saturated/total fat ratio.

The distributions of diet variables by quintiles of CFr, eCarb, and eFat are illustrated by box plots in Figure 3. For CFr (Figure 2A), the distributions of eProt, eAlc, and SAFr were very homogenous between all quintiles. Comparisons of extreme quintiles indicated that eCarb (Figure 2B) and eFat (Figure 2C)—compared with CFr*—*were substantially more imbalanced regarding other diet variables. That conclusion, on the basis of graphical interpretations, was further confirmed by a comparison of correlations, for example, eCarb–eProt (*r* = −0.33) and eCarb–eAlc (*r* = −0.37), whereas corresponding numbers for CFr were −0.12 and −0.08. Further characteristics by diet quintiles (CFr, eProt) are shown in Supplemental Figure 2.

In 0.5% of the observations eCarb was ≤25 E%, that is, in the range often defined as low-carbohydrate [42]. Reported intake of eAlc was 0 for 61% of the observations, with percentile 75 and 90 being 1.6 and 5.5 E%, respectively. Beyond eAlc, distributions of the diet variables were fairly normal. For participants in the lowest 10 percentiles of cognitive progression (Δ-score y 0–2) per group, neither levels (*P* = 0.94) nor change (*P* = 0.20) of CFr differed significantly from the rest of the sample.

### Estimates between diet and cognition

**FIGURE 4:**
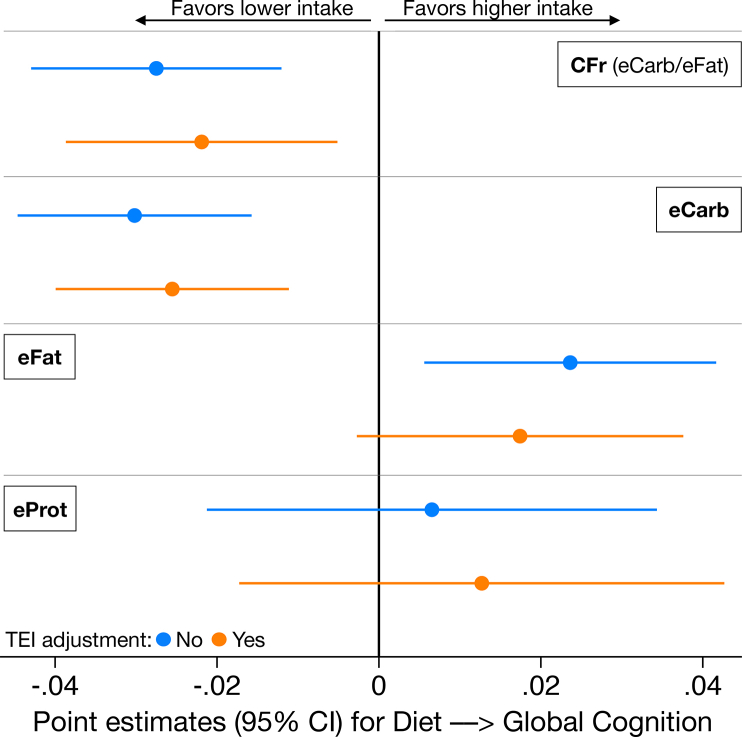
Primary estimates for diet variables as predictors of global cognition. Models without (blue) and with (orange) adjustment for TEI (standardized by sex). Linear mixed regression adjusted for age, sex, education, BMI, use of statins or other cholesterol-lowering drugs, time, time × group, and clustering on study center and subject (*n* = 1247; data on diet and cognition from years 0, 1, and 2). CFr, log-ratio carbohydrates/fat; eCarb, carbohydrates by E%; eFat, fat by E%; eProt, protein by E%; TEI, total energy intake. Diet and cognitive variables measured on a *z*-scale.

In Table 2, results for CFr, eCarb, eFat, and eProt as predictor variables of global cognition are reported. Those estimates are additionally shown graphically in Figure 4 to facilitate a descriptive comparison of eCarb and eFat in relation to CFr. Very small differences were observed between models, regardless of included covariates or whether performed as sensitivity analyses including only participants with complete data (Supplemental Figures 3 and 4). For model A (crude, *P* = 0.001 for CFr → global cognition), the magnitude of the point estimate was reduced by 4%, and for model B (age, sex, education, *P* = 0.002), the reduction was 8%, compared with model C in matched samples (*n* = 1028). The inclusion of SAFr and eFib as covariates or interaction terms had negligible impact on the CI for CFr [and eFib itself was not associated with global cognition (*β* = 0.00, *P* = 0.95)]. The CI for *CFr* was likewise negligibly affected by adjustment for eProt or FDAS. Therefore, we restrict the further reporting to 2 models: *1*) model C, with age, sex, education, BMI, cholesterol-lowering drugs, time, and time × group as covariates. *2*) Additional adjustment for TEI.

**TABLE 2:** Estimates between diet variables and global cognition

Predictor variable	Adjusted for age, sex, education, BMI, and statins		Additional adjustment for TEI	
	*β* (95% CI)	*P* value	*β* (95% CI)	*P* value
CFr	−0.027 (−0.043, −0.012)	<0.001	−0.022 (−0.039, −0.005)	0.011
eCarb	−0.030 (−0.045, −0.016)	<0.0001	−0.026 (−0.040, −0.011)	<0.001
eFat	0.024 (0.006, 0.042)	0.010	0.017 (−0.003, 0.038)	0.090
eProt	0.007 (−0.021, 0.034)	0.64	0.013 (−0.017, 0.043)	0.41

Abbreviations: CFr, log-ratio carbohydrates/fat; eCarb, digestible carbohydrates by E%; eFat, total fat by E%; eProt, protein by E%; TEI, total energy intake.

Mixed effects linear regression using data on diet and cognition from year 0, 1, and 2, adjusted for time, time × randomization group, plus indicated covariates, with study site and subject as random factors. Statins refers to use of any cholesterol-lowering drug. Diet and cognition measured on a *z*-scale (*n* = 1247).

**FIGURE 5:**
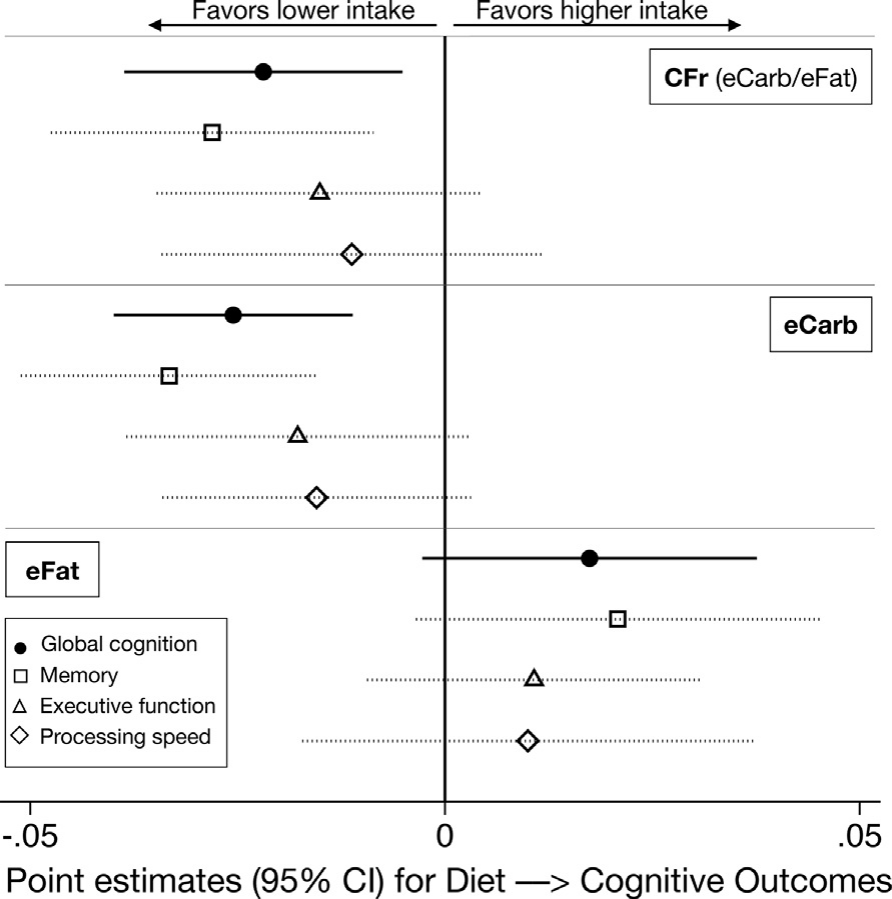
Estimates between diet variables and cognitive subdomains. Linear mixed regression adjusted for age, sex, education, BMI, use of statins or other cholesterol-lowering drugs, TEI (standardized by sex), time, time × group (*n* = 1247; data on diet and cognition from years 0, 1, and 2). CFr, log-ratio carbohydrates/fat; eCarb, carbohydrates by E%; eFat, fat by E%. Diet and cognitive variables measured on a *z*-scale.

We proceeded with investigating how the associations for CFr, eCarb, and eFat were reflected in the cognitive subdomains, that is, memory, executive function, and processing speed (Figure 5), using the model adjusted for TEI. As indicated, the direction of the association with cognition was consistent between the subdomains, with the greatest magnitude for memory (CFr: *β* = −0.028, CI: −0.048, −0.009; *P* = 0.005).

**FIGURE 6:**
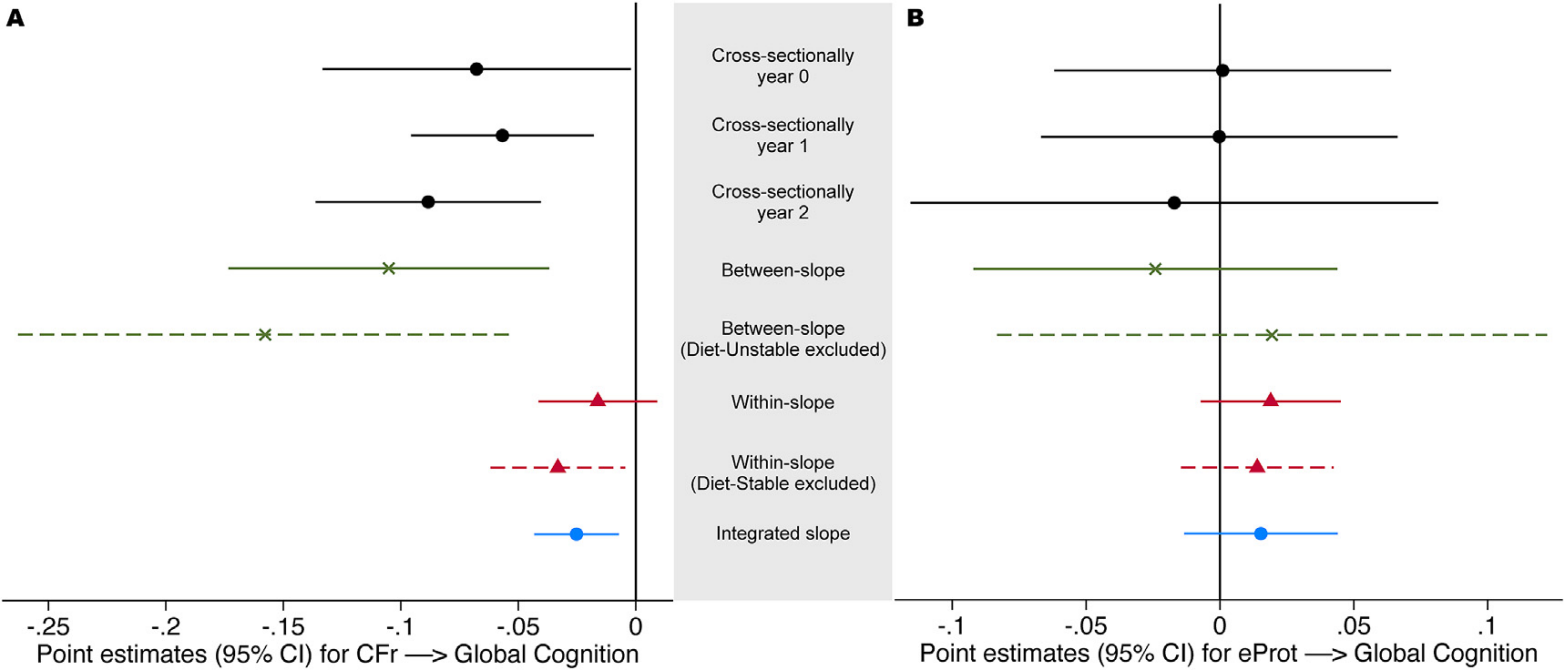
Complementary regression analyses between diet variables and global cognition. (A) The log ratio carbohydrates/fat (CFr) and (B) protein by E% (eProt) as predictor variables in regression models adjusted for age, sex, education, BMI, cholesterol-lowering drugs, TEI (standardized by sex), time, time × group, and clustering on study site and subject. Matched samples with complete data (*n* = 947). Cross-sectional analyses (black) use data from a single year; the other analyses use data from all 3 y. The between-subject slope (green cross) is estimated by collapsing each subjects’ repeated measures per variable to a mean. The within-slope (fixed effects model, red triangles) is based on intraindividual variability in relation to intraindividual means. The model in blue is the primary estimate (mixed effects model), used in Figure 4. Diet-stable/unstable defined by median-split, on the basis of intraindividual difference between highest and lowest value of each diet variable among the repeated measures (see Figure 1B). Those sensitivity analyses (striped) were assumed to restrict the samples to participants where the slopes most reliably represent long-term diet (between) and “true” dietary changes (within), respectively. Diet and cognitive variables measured on a *z*-scale.

Complementary analyses are reported in Figure 6, illustrating consistency over time points for the cross-sectional association between diet variables and cognition, and coherence of the subcomponents of the slope reported in Figure 4. There was no interaction by group in the cross-sectional associations at year 1 and 2 for CFr (*P* > 0.39) or eProt (*P* > 0.94). As an additional analysis of consistency, interactions diet_variable × time → global cognition in the mixed model showed no significance for CFr (*P* = 0.90) or eProt (*P* = 0.81), and neither did interactions by diet_variable × time × group (CFr: *P* = 0.88; eProt: *P* = 0.14).

The estimated difference in cognition between CFr at 2 SD below and above the mean—corresponding to 34:45 compared with 59:20 [crude estimates eCarb:eFat (E%)]—yielded a difference in global cognition of 0.087 (*z*-scale) in favor of a lower CFr for the mixed model. In comparison, the within-subject estimate was 22% lower and the between-subject estimate was 275% higher. As a reference, the lower CFr value corresponds closely to means of the intervention groups in the PREDIMED trial [43], whereas the higher value coincides with the intervention target for the Women’s Health Initiative [18].

**FIGURE 7:**
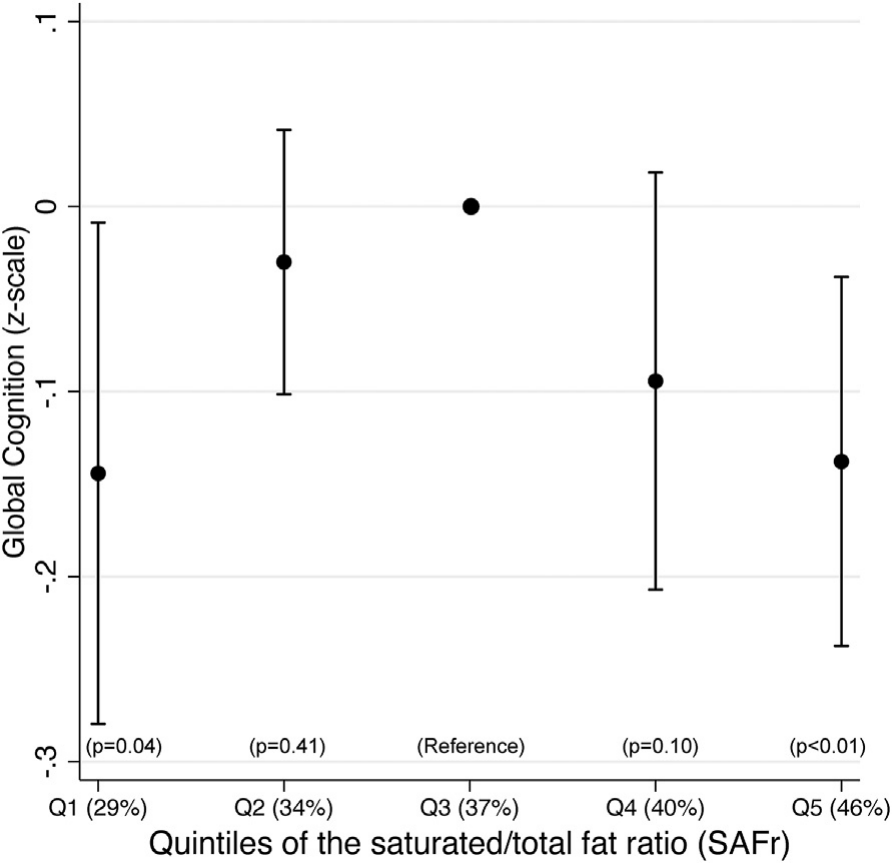
The saturated/total fat ratio (SAFr) and global cognition. Cross-sectional baseline analysis on the SAFr by quintiles as a predictor of global cognition (*n* = 1199). Mixed regression adjusted for age, sex, education, BMI, use of cholesterol-lowering drugs, TEI (standardized by sex), and clustering on study site. Mean SAFr (as %) per quintile (Q) is indicated.

Because observed nonlinearity for SAFr prohibited its inclusion in FIGURE 4, FIGURE 5, FIGURE 6, we report an exploratory cross-sectional baseline analysis by quintiles in Figure 7, confirming significantly lower cognitive performance in the extreme quintiles (SAFr ranges Q1: 20.3%–32.0%; Q5: 42.3%–59.2%) compared with the mid-quintile (Q3: 35.6%–38.8%). Because of missing covariate data, the sample was reduced to *n* = 1199 for that analysis. There was substantial individual movability between quintiles over time points and at year 2 (*n* = 1039), only Q5 had substantially lower cognitive performance than Q1–4 (*β* = 0.32, 0.35, 0.28, 0.31, all *P* < 0.014), whereas none of Q1–4 differed between each other (all *P* > 0.42). The longitudinal within-subject slope for SAFr in relation to global cognition indicated no trend (*β* = 0.00, *P* = 0.81).

## Discussion

### Estimates of cognitive performance for CFr, eCarb, and eFat

In this study, we found significant associations between cognitive performance and self-reported intake levels of eCarb, eFat, and their ratio CFr among older adults with risk factors for dementia. CFr and eCarb were negatively associated with global cognition. The slope for eFat was positive, but significant only without adjustment for total energy intake (TEI). The relations were most pronounced in the memory subdomain, which is interesting considering its key role in Alzheimer’s disease [44]. To our knowledge, this is the first report on estimates between macronutritional composition and cognitive performance in a sample of older adults selected by risk factors for dementia, and the first report with CFr as an explicit predictor of a cognitive health outcome. Because the sample represents a key target population for preventive lifestyle interventions, the observations are interesting and warrant further studies.

The magnitudes of the point estimates were eCarb>CFr>eFat, but CFr appeared as the most independent predictor variable by having lower correlations with other diet variables. Regardless of which of those variables was chosen as the base for quintiles, the distributions of eCarb, eFat, and CFr were very similar in the extreme quintiles (Figure 3), and the differing point estimates might be explained by differing distributions of outliers in eProt and eAlc, which may confound the results more for eCarb and eFat than for CFr. Our data suggest that those 3 variables may represent 1 dimension in macronutritional composition: CFr. When correlation exceeds *r* > |0.7|, it has been recommended that collinear variables should not be used in the same model but preferably be reduced to 1 variable by various strategies [45,46]. We conclude that the informative value of including eCarb and eFat (which correlate by *r* = −0.74 internally, and by *r* = 0.92/−0.94 with CFr) as separate predictors does not outweigh the induced collinearity and multiple-comparisons issue, and therefore CFr could be considered an alternative predictor—which in this sample was demonstrated to approximate isocaloric replacement between eCarb and eFat.

The complementary analyses on CFr were coherent between time points cross-sectionally, and for the between- and within-subject slopes, although the within-subject slope was not significant in the full sample. The overall lower magnitude of the within-subject slopes compared with the between-subject slopes might reflect the shorter timespan they possibly represent cumulatively or differentiating confounding: an advantage of the within-subject slope is the absence of confounding by measured and unmeasured time-invariant variables [30], for example, traits such as “health awareness.” The unsystematic distribution of each subject’s highest/lowest/mid-intake over time points (Figure 1) makes reverse causation unlikely, in line with the observations that mean levels or changes in CFr were not different among participants with lowest 10 percentiles of cognitive progress.

The results for CFr were remarkably stable when various covariates—including fiber intake and the proportions of fat-subtypes (SAFr)—were added. TEI (which correlated *r* = −0.15 with CFr) was the most influential covariate and reduced the magnitude of the estimate CFr → Cognition by ≈20%. This might reflect confounding, for example, as a proxy for physical activity level [47]. Because the compositional aspect of diet (CFr) might have a causal effect on the quantitative aspect (TEI) [5], it could alternatively reflect mediation, and if so the unadjusted (blue) estimates of Figure 4 might be the least biased.

Among observational studies with dementia, mild cognitive impairment, or cognitive decline as the outcome, we only identified 3 studies reporting estimates from eCarb intake: favoring lower [23], favoring higher [19], or reporting no association [21]. Those analyses compared quantiles by E% with the lowest quantile covering eCarb ≤ 47 E%, indicating that any conclusion on low-carbohydrate diets (≤25 E% [42]) cannot be made by current evidence. Several studies with total fat as the independent variable found no relationship [[20], [21], [22],^24^], whereas a United States longitudinal study favored higher [23] and a Chinese cross-sectional study favored lower [19] intake. Those studies primarily applied various quantiles, and either analyzed fat by grams or E%. A review from 2018 [3] identified no cognitive RCT related to eCarb or eFat, and we have only found 1 more recent such publication with a diagnosis of dementia or cognitive impairment as a reported outcome: no significant differences on those outcomes between the intervention group (low-fat + increased intake of fruits, vegetables, and grains) and the control group (usual diet) were found [18]. It may further be relevant to notice that in 3 cognitive RCT supporting the Mediterranean diet [43,48,49], the control group had a higher CFr. Moreover, a pilot trial (6 + 6-wk, cross-over) reported some improved Alzheimer biomarkers on a Modified Ketogenic Mediterranean diet (very-low-CFr) compared with a high-carbohydrate-low-fat diet among individuals with subjective or mild cognitive impairment [50].

### Estimates of cognitive performance for protein and the saturated/total fat ratio

Protein intake was not significantly associated with global cognition in these analyses, which is in line with a recent meta-analysis on the association between protein intake and cognitive function in older adults [51]. Patterns of relatively higher intake of protein compared with carbohydrates were associated with lower risk of cognitive decline in 2 United States samples [23,52], whereas the opposite association was observed in a Chinese sample [19].

We did not find a linear association between SAFr and cognition. At baseline, the mid-quintile (36%–39%) had better cognitive performance compared with the lowest as well as the highest quintile. A cross-sectional analysis at year 2 found negligible differences between Q1 and Q4 but substantially lower cognition for Q5 compared with the others. No trend was observed in the longitudinal within-subject slope between SAFr and cognition.

We have not identified any previous cognitive study reporting SAFr, nor the almost perfectly correlated ratio eSFA/(eMUFA + ePUFA). Some studies reported SFA as a ratio of either PUFA or (inversely) MUFA, including positive [53], negative [54], and neutral [55] associations for higher intake. A recent review—commissioned by the committee for updating NNR—found no robust association between intake of SFA and risk of Alzheimer’s disease or dementia, and the authors called for more well-designed prospective studies [56]. That is in line with another recent review that found no association between intake of SFA (or total fat) and MCI/Alzheimer/dementia [57]. Astrup et al. [58] pointed out that health effects of saturated fat intake may depend on the actual food source, heterogeneity within the category SFA, and on individual differences in biomarker responses. Such factors might underly inconsistencies in the cognitive literature and in the results for SAFr within our study. Considerations of this kind of factors should facilitate the interpretation for all diet variables.

### Limitations and strengths

A limitation of this study is that the observations were made in the context of an ongoing multidomain lifestyle intervention. However, the changes in mean CFr were not different between the intervention and control groups, and CFr decreased only slightly over time. There was an overall increase in fat intake in the Finnish population during the study period that may correspond to those changes over time. The results remained stable even after adjustment for many potential confounders, but we cannot exclude unmeasured confounding. Furthermore, bias might have occurred in capturing the habitual food intake using data from the 3-d food records. The directed selection of this sample [33] reduces generalizability of the results to the general population and replications of the analyses among various samples are warranted. We did consider collider bias related to the inclusion criteria [59] but found it unlikely that diet exposure would be substantially biased. A strength of this study is the rigorous and extensive data collection, including repeated measures of both the independent and the dependent variables. Furthermore, we addressed methodological issues related to the high correlations between some macronutrients. The ratios CFr and SAFr correspond to well-defined compositional research questions, and the advantages of using ratios in nutrition research have been described by others [60,61].

By retrospectively following suggestions [62] to define the target trial of observational studies, our within-slope may represent a cross-over trial where participants are assigned to (relative) low-mid-high levels of the diet parameter for <1 y each (in a fairly “randomized” order according to Figure 1). The between-slope should represent a parallel design over several years (and thereby a possible larger cumulative effect). The exclusion of diet-stable (less meaningful contrast within) and diet-unstable (less reliable long-term proxy for between) in Figure 6 may mimic a per protocol analysis [63]. Estimates of the mixed model appeared to fall closer to the within compared with the between-slope.

In conclusion, this study found that a lower dietary CFr was associated with better global cognition in older adults at risk for dementia. High-quality RCTs are warranted to better understand the possible impact of carbohydrate reduction on cognitive health.

## Funding

This work was supported by a grant from the af Jochnick Foundation, and from the following funding sources: MK receives support from the Swedish Research Council, Alzheimerfonden, Center for Innovative Medicine at Karolinska Institutet South Campus, Knut and Alice Wallenberg Foundation, Stiftelsen Stockholms Sjukhem (Sweden), Hjärnfonden, ALF, and FORTE. SS receives research support from Alzheimerfonden, Demensfonden, and Karolinska Institutet Research Foundation Grants (KI Fonder). The Finnish Geriatric Intervention Study to Prevent Cognitive Impairment and Disability trial data collection was supported by the Academy of Finland, Juho Vainio Foundation (Finland), Kela (Finland), Alzheimer’s Research and Prevention Foundation (United States), Finnish Cultural Foundation, and Yrjö Jahnsson Foundation. The funding sources were not involved in the content or publication of the article.

## Author disclosures

MK reports the following relations: Advisory board membership for BioArctic, Biogen, Combinostics, and Nestlé and speaking and lecture fees for Biogen, Nestlé, Nutricia, and Roche. JN, SS, ASM, TN, and IK, no conflict of interest.

## Data availability

Public deposition of the deidentified data set is not possible due to legal and ethical reasons, and complete deidentification is not possible because this investigation is part of an ongoing study. The study participants gave informed consent that includes data use only under confidentiality agreement. Furthermore, the data contain large amount of sensitive information, and public data deposition may pose privacy concerns. Data dictionary relevant for the present study can be shared upon request to the Finnish Institute for Health and Welfare (THL): kirjaamo@thl.fi. Pseudonymized personal data relevant for the present study can be made available only for those fulfilling the requirements for viewing confidential data as required by the Finnish law and THL. Data will be made available only for the purpose of research that is in alignment with informed consent, with investigator support and after approval of a proposal and completion of material transfer agreement.

## Appendix A Supplementary data

Multimedia component1 Multimedia component1
