# Supplementary material for: The Dietary Carbohydrate/Fat-Ratio and Cognitive Performance: Panel Analyses in Older Adults at Risk for Dementia
Source: Curr Dev Nutr. 2023 May 7;7(6):100096. doi: 10.1016/j.cdnut.2023.100096 (PMC10236460; doi:10.1016/j.cdnut.2023.100096)
Supplement: Multimedia component1 [file mmc1.pdf]

The Dietary Carbohydrate/Fat-ratio and Cognitive Performance: Panel Analyses in Older Adults at Risk for Dementia

(Norgren et al.)

Online Supplementary Material

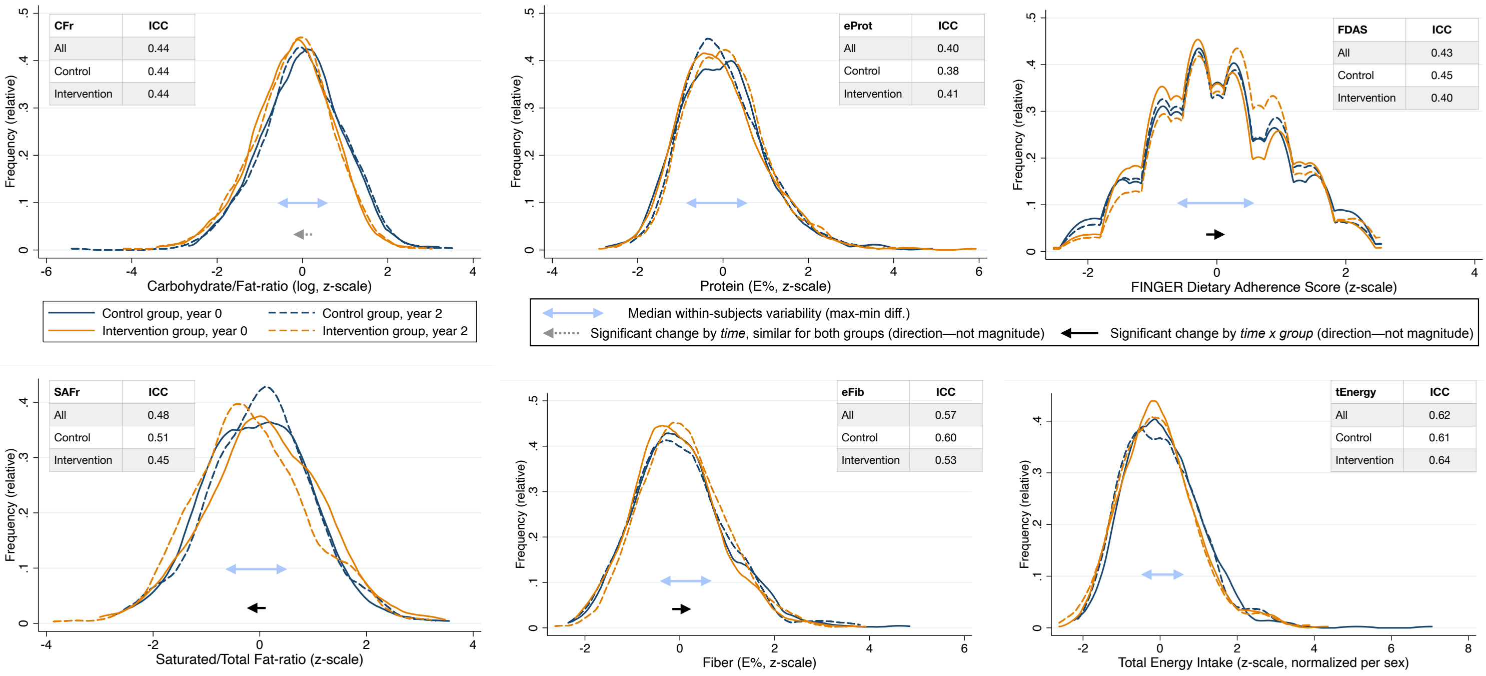

Supplementary Figure 1. Variability in diet variables by time and randomization group
Homogenous ranges of distribution illustrated for all variables. When mean levels differ significantly by *time* (gray arrow) or *time x group* (black arrow, intervention vs. control) it is indicated by arrows representing direction—not magnitude. Blue arrows represent magnitude of the median intra-individual difference between each subjects highest and lowest measure, independent of order. The lengths of those arrows are intended to roughly correspond to the following values on a z-scale: 1.1 (CFr, protein, SAFr), 0.9 (fiber, tEnergy), 1.3 (FDAS). Year 1 excluded to enhance visibility but would not change any conclusions. tEnergy standardised by sex. ICC: Intra-class correlation coefficient

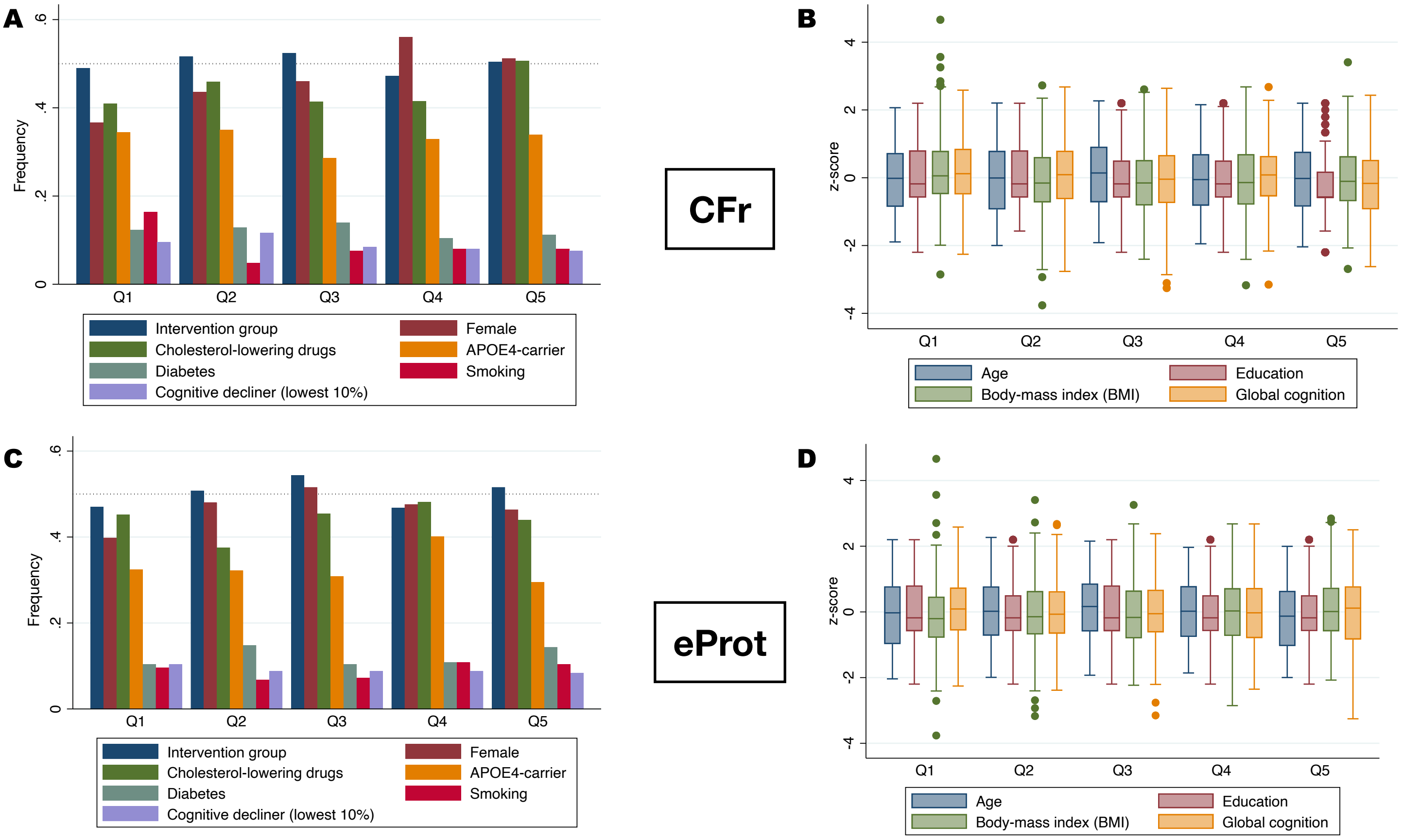

Supplementary Figure 2. Characteristics per diet quintile
Distributions of characteristics over diet quintiles for the carbohydrate/fat-ratio (CFr, panel **A-B**) and protein (eProt, panel **C-D**). Baseline values, except “Cognitive decliner” which represents being in the lowest 10 percentiles per group in change in global cognition between years 0 and 2. Boxes indicate percentiles 25, 50, 75.

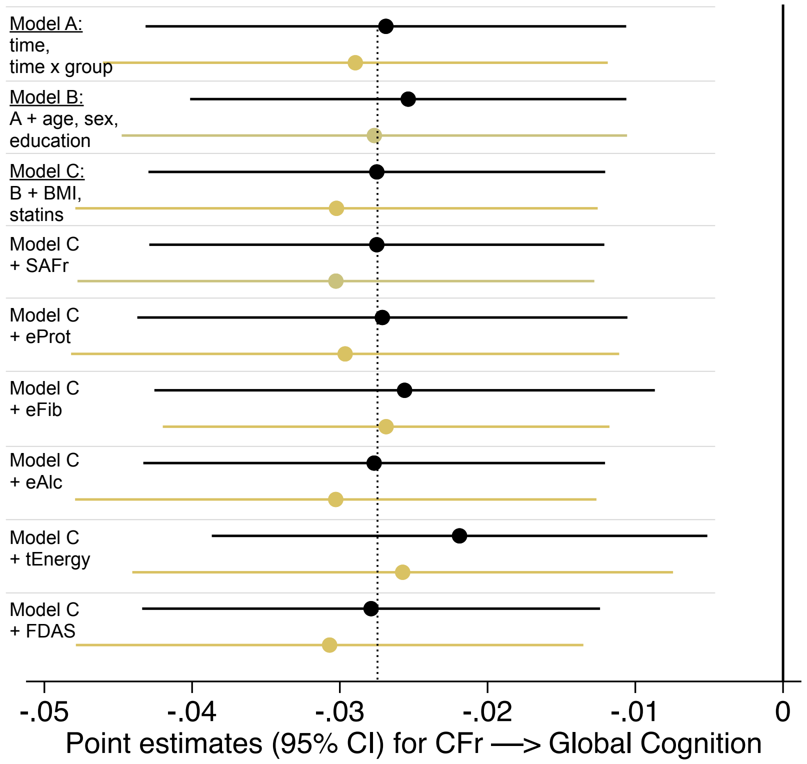

Supplementary Figure 3. Estimates for the carbohydrate/fat-ratio (CFr) as a predictor of global cognition.
BLACK: n=1247; SAND: n=1028, with complete data from all timepoints. Mixed regression using data from yer 0, 1 and 2 with study site and subject as random factors. BMI: body-mass index, statins: statins or other cholesterol-lowering drug; eAlc: alcohol; eFib: fiber; eProt: protein (prefix e: by E%), FDAS: FINGER dietary adherence score; SAFr: saturated/total fat ratio, tEnergy: total energy intake standardised per sex. Dotted reference line for Model C.

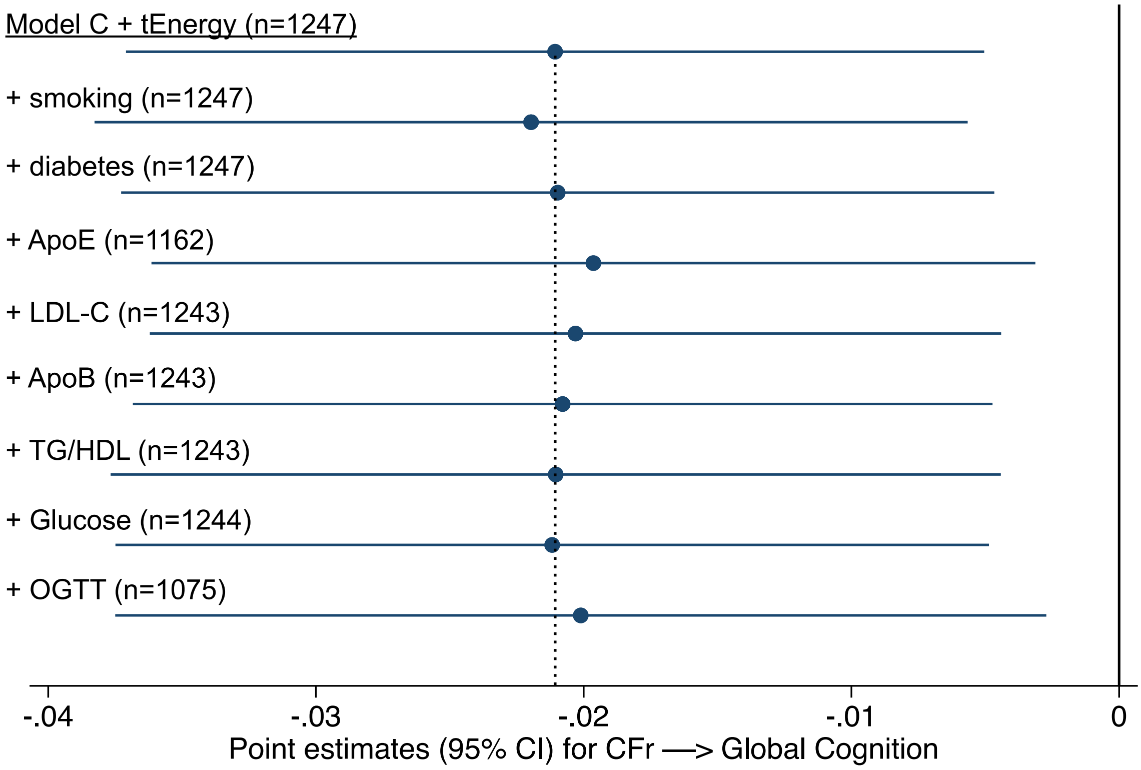

Supplementary Figure 4. Estimates for the carbohydrate/fat-ratio (CFr) as a predictor of global cognition.
Baseline characteristics added as covariates one at the time to Model C (adjusted for age, sex, education, body-mass index, use of cholesterol lowering drugs, time, time x group) + total energy intake standardised per sex (tEnergy). Mixed regression using data from year 0, 1 and 2 with study site and subject as random factors. ApoE: apolipoprotein E genotype, LDL-C: low-density lipoprotein cholesterol, ApoB: apolipoprotein B, TG/HDL: ratio triglycerides / high-density lipoprotein cholesterol, Glucose: fasting levels, OGTT: oral glucose tolerance test (glucose at 120 min.) Log-transformation was applied to ApoB, TG/HDL, glucose, and OGTT. Dotted reference line for Model C + tEnergy.
